# Relationship Between Brachial Artery Flow-Mediated Dilation, Carotid Artery Intima-Media Thickness and Coronary Flow Reserve in Patients With Coronary Artery Disease

**DOI:** 10.4021/cr219w

**Published:** 2012-09-20

**Authors:** Fahrettin Oz, Ali Elitok, Ahmet Kaya Bilge, Fehmi Mercanoglu, Huseyin Oflaz

**Affiliations:** a Department of Cardiology, Istanbul University, Istanbul Faculty of Medicine, Istanbul, Turkey

**Keywords:** Endothelial dysfunction, Atherosclerosis, Coronary artery disease, Coronary flow reserve, Flow mediated dilation, Carotid artery intima-media thickness

## Abstract

**Background:**

The aim of this study was to assess the relationship between brachial artery flow mediated dilation (FMD), carotid artery intima-media thickness (IMT) and coronary flow reserve (CFR) in patients with coronary artery disease (CAD).

**Methods:**

Fifty patients with coronary artery disease, except left anterior descending artery (LAD), who showed no cardiac symptoms and 45 control subjects underwent assessment of brachial artery FMD, carotid artery intima-media thickness by high-resolution ultrasound. In addition, transthoracic second harmonic Doppler echocardiography was used to measure CFR.

**Results:**

All of the parameters were found to be correlated with each other. CFR correlated with brachial artery FMD (r = 0.232, P < 0.05) and with carotid IMT (r = -0.403, P < 0.001). Carotid IMT correlated with brachial artery FMD (r = -0.211, P < 0.05).

**Conclusion:**

Transthoracic CFR correlated with well-established noninvasive predictors of atherosclerosis and we suggest that it can be used as a surrogate for coronary atherosclerosis.

## Introductıon

Endothelial dysfunction, by impairing vasomotor tonus, promoting arterial thrombosis, vascular cell migration and proliferation is a major determinant of atherosclerosis [1, 2]. Endothelial dysfunction is an early and reversible key event of cardiovascular disease and has been used to predict future coronary artery disease prior to atherosclerotic changes in arteries has occured [1]. Inflammation has a critical role in the pathogenesis of atherosclerosis and endothelial dysfunction [1, 2]. Coronary atherosclerosis is often clinically silent, with death as its first manifestation. Much effort has been made to detect the beginning of the disease. Because primary risk reduction is effective, several noninvasive options to assess atherosclerosis in its preclinical stages have been introduced into clinical use. These include carotid artery intima-media thickness (IMT), brachial artery flow-mediated dilation (FMD). Recently transthoracic second harmonic Doppler echocardiography has evolved as a tool to investigate coronary flow pattern and coronary flow reserve (CFR). Endothelial dysfunction precedes clinically manifested atherosclerosis and its assessment through the use of high-resolution ultrasound to measure brachial artery diameter in response to reactive hyperemia has increased the clinical relevance of vascular function evaluation [3]. High-resolution ultrasound provided evidence that reduced endothelium-mediated vasodilation is associated with increased risk for cardiovascular events in a variety of conditions [4]. In patients with coronary artery disease endothelium-mediated vasodilation of the brachial artery correlates with the coronary vasodilator response [5]. Normally coronary blood flow can increase approximately four-to-six fold to meet increasing myocardial oxygen demands. This effect is mediated by vasodilation at the arteriolar bed, which reduces vascular resistance, thereby augmenting flow. The coronary reserve represents the capacity of the coronary circulation to dilate, following an increase in myocardial metabolic demands and can be expressed by the difference between the hyperemic flow and the resting flow [6]. Coronary flow reserve which depends on endothelium mediated vasoreactivity, also may be impaired in the absence of manifested CAD, and the evaluation of CFR may allow early detection of coronary atherosclerosis [6]. Although CFR was used to be measured invasively until recently, CFR has been evaluated in echo-lab by using Doppler and vasodilator stress such as dipyridamole or adenozine [6]. By this method, impairment of CFR can be assessed before development of angiographically detectable stenosis in epicardial coronary arteries and we are able to investigate early coronary microvasculature pathology [7]. Measurement of CFR has predictive power for future cardiovascular events and therefore could be used as a surrogate marker for early atherosclerotic changes of coronary arteries [6, 7].

## Methods

We studied 50 patients (40 men; mean age 52.4 ± 6.4 y) who had evidence of CAD at coronary angiography. Fortytwo patients underwent coronary angiography because of stable angina pectoris and 8 in consequence of prior myocardial infarction (at least 1 year ago and not anterior MI). Forty five subjects (36 men; mean age 49.8 ± 8.9 y) who did not have structural heart disease and who were referred for functional evaluation of chest pain represented the control group. Control group subjects were evaluated with treadmill exercise electrocardiogram testing and none of them were excluded owing to ischemic ECG changes in response to exercise.

All CAD patients and control subjects underwent evaluation after withdrawal of all cardiac drugs for ≥ 18 h before the study. All abstained from smoking and intake of caffeine-containing food for at least 24 h before the study. The ethical committee approved the protocol, and each patient or control subject gave informed consent.

### FMD evaluation by brachial artery high-resolution ultrasound imaging

Flow mediated dilation was measured according to recent guidelines [8] with ultrasound unit electronic calipers (VIVID 7 General Electric, Wisconsin, and USA) and 10 MHz linear array transducer. To best visualize the brachial artery, the arm was comfortably immobilized in the extended position and the brachial artery was scanned in a longitudinal section 3 - 5 cm above the antecubital fossa. After optimal transducer positioning, the skin was marked for reference for later measurements. Three consecutive measurements obtained through consecutive cardiac cycles were averaged and recorded. Briefly, FMD was assessed by measuring the change in brachial artery diameter after 60 s of reactive hyperemia compared with a baseline measurement after deflation of a cuff that had been placed around the forearm and that had been inflated to 50 mmHg above systolic blood pressure for 5 min. The response of the vessel diameter to reactive hyperemia was expressed as the percent change relative to the diameter immediately before cuff inflation. Flow mediated dilation was expressed as the percentages change in the brachial artery internal diameter from baseline following reactive hyperemia.

### Measurements of carotid intima-media thickness

Carotid arteries provide a window to the coronary arteries. Patients with major carotid stenosis are very likely to have major coronary stenosis. Measurements of carotid IMT with ultrasound is a noninvasive and highly reproducible technique for quantifying atherosclerotic burden. That is the relationship between the atherosclerotic burden in a carotid artery and coronary artery is the same as between any two coronary arteries [9]. Considering the high correlation between carotid and coronary artery disease, carotid screening is useful in patients with coronary artery disease. In patients with an occasional finding of a carotid risk score of at least 2 (IMT more than 0.90 mm, unstable plaque and severe stenosis > 70%) a careful search for coronary artery disease seems warranted [10]. Carotid IMT has predictive power for future myocardial infarction and stroke beyond the well-known coronary risk factors [11, 12]. Accordingly American Heart Association (AHA) prevention conference has recommended carotid IMT scanning for patients older than 45 years who require further clarification of their coronary heart disease risk [12]. In this study carotid IMT was measured according to the method described previously [9, 11]. With the subject in the supine position, longitudinal scanning was performed from the common carotid artery to the bifurcation point. After the bifurcation of the common carotid artery had been confirmed, carotid IMT was measured from the far wall of the internal carotid artery within 10 mm proximal to the bifurcation. Three points were measured on one scan, which was synchronized with R wave peaks on the ECG to avoid possible errors resulting from variable arterial compliance. Mean carotid IMT was calculated from six measurements on two scans.

### Transthoracic coronary flow measurement

Coronary flow reserve measurements are used to assess epicardial coronary arteries and to examine the integrity of coronary microvascular circulation. In recent years, transthoracic second harmonic Doppler echocardiographic examination of CFR has become very populer and its feasibility has been validated [13-15]. In this study CFR recordings were performed with the Vivid 7 echocardiography device (General Electrics, Wisconsin, and USA) using a middle-range frequency (3 - 8 MHz) broadband transducer. Visualization of the distal LAD was performed using a modified, foreshortened; two chamber view obtained by sliding the transducer on the upper part and medially, from two-chamber view. Subsequently, coronary flow in the distal LAD was examined by color Doppler flow mapping over the epicardial part of the anterior wall, with the color Doppler velocity range set in the range of 9 - 24 cm/sn. The acoustic window was around the midclavicular line, in the fourth and fifth intercostal spaces, with the subject in the left lateral decubitus position [16, 17]. By placing the sample volume on the color signal, spectral Doppler of the LAD showed the characteristic biphasic flow pattern, with larger diastolic and smaller systolic components. After baseline recordings of flows, dipyridamole (0.56 mg/kg, Persantin, Boehringer Ingelheim, Barcelona, Spain) was infused over a 4-min. period. Two minutes after the end of the infusion, hyperemic spectral profiles in the LAD artery were recorded. By averaging the three highest Doppler signals for each measurement, CFR was defined as the ratio hyperemic to baseline diastolic peak velocities. CFR > 2 was considered normal [16, 17].

### Statistical analysis

Continuous data were expressed as mean ± SD, and categorical data were expressed as percentages. Chi square-test for categorical variables was used to assess differences among groups. The relationships among parameters were assessed by pearson correlation analysis. A P value of < 0.05 was considered statistically significant.

## Results

**Figure 1:**
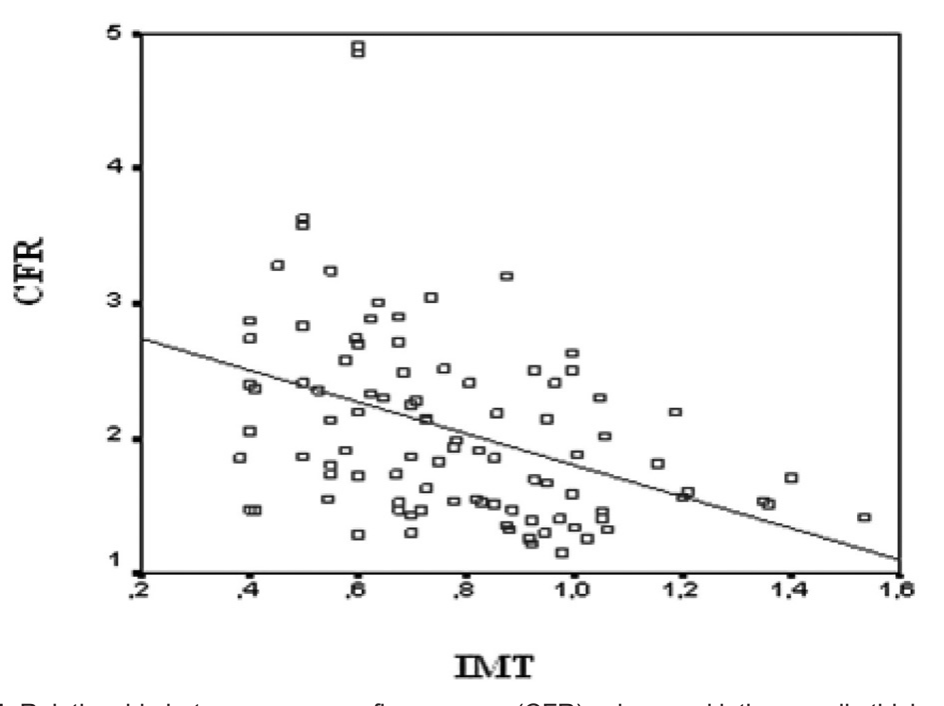
Relationship between coronary flow reserve (CFR) values and intima-media thickness of all subjects in study group (r = -0.403, P < 0.001).

**Figure 2:**
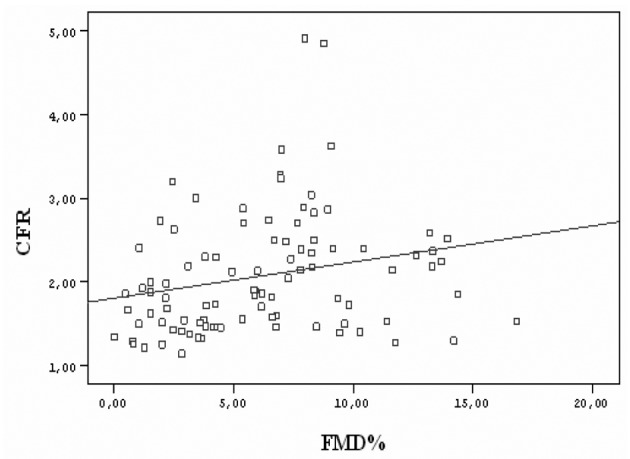
Relationship between coronary flow reserve (CFR) values and flow mediated dilation (FMD) of all subjects in study group (r = 0.232, P < 0.05).

**Figure 3:**
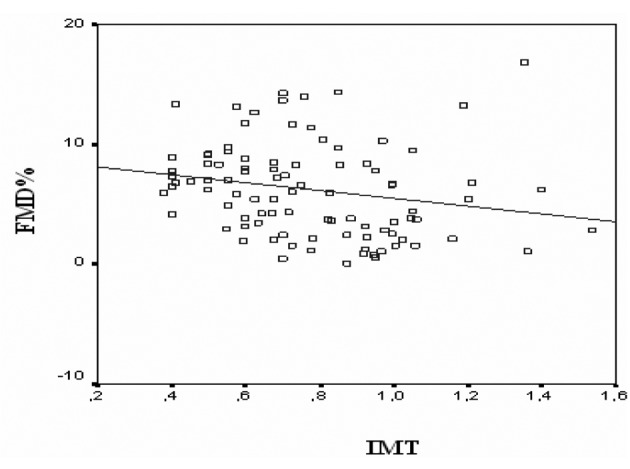
Relationship between flow mediated dilation (FMD) and intima-media thickness (IMT) of all subjects in study group (r = -0.211, P < 0.05).

All CAD patients and control subjects successfully completed the dypridamole perfusion study without major side effects. Individual characteristics, FMD, IMT and CFR for patients with CAD and for control subjects are reported in Table 1, 2, respectively. Flow mediated dilation was 7.47 ± 2.94 % in control subjects and 5.03 ± 4.24 % in CAD patients (P = 0.02). Intima-media thickness was 0.64 ± 0.17 in control subjects and 0.89 ± 0.24 in CAD patients (P < 0.001). Coronary flow reserve was 2.40 ± 0.80 in control subjects and 1.75 ± 0.46 in CAD patients (P < 0.001). All of the parameters correlated with each other. Coronary flow reserve correlated with carotid IMT (r = -0.403, P < 0.001) (Fig. 1) and brachial artery FMD (r = 0.232, P < 0.05) (Fig. 2). Carotid IMT showed negative correlation with brachial artery FMD (r = -0.211, P < 0.05) (Fig. 3).

**Table 1:** Clinical Characteristics of the Subjects and CAD Patients

	CAD (n = 50)	Control (n = 45)	P
Age (year)	52.4 ± 6.4	49.8 ± 8.9	NS
Male/Female	40/10	36/9	NS
BMI (kg/m^2^)	26.2 ± 3.44	26.1 ± 2.94	NS
Hypertension	32 (64%)	9 (20%)	< 0.05
Diabetes Mellitus	7 (14%)	-	
Smoke	37 (74%)	30 (66.7%)	NS
Systolic pressure (mmHg)	126.9 ± 15.76	123 ± 17.16	0.39
Diastolic pressure (mmHg)	79.2 ± 9.64	77.44 ± 8.38	0.19
Total Chol (mg/dL)	186.9 ± 41.5	195.4 ± 30.8	NS
Triglyceride (mg/dL)	158.18 ± 84	138.08 ± 38.51	NS
HDL (mg/dL)	38.24 ± 8.71	42.75 ± 6.06	< 0.05
LDL (mg/dL)	112.04 ± 34.69	130.02 ± 27.78	< 0.05
Creatinin (mg/dL)	0.94 ± 0.23	0.83 ± 0.13	NS
Hgb (g/dL)	13.7 ± 1.03	14.09 ± 1.61	NS
Medical Treatment			
ACE inhibitor	35 (70%)	5 (20%)	< 0.001
Beta blocker	37 (74%)	3	< 0.001
Calcium C. blocker	2 (4%)	1	NS
Aspirin	48 (96%)	2	< 0.0001
Statin	45 (90%)	1	< 0.0001

NS: not significant; BMI: Body-Mass Index.

**Table 2:** Echocardiographical Features of the Control and the CAD Patients

	CAD (n = 50)	Control (n = 45)	P
EF (%)	56% ± 7.33	64% ± 4.59	< 0.05
LVMI (g/m^2^)	109 ± 25.48	85.74 ± 16.91	< 0.005
IMK (mm)	0.89 ± 0.24	0.64 ± 0.17	< 0.005
FMD (%)	5.03 ± 4.24	7.47 ± 2.94	= 0.02
BPDV (cm/sn)	32.68 ± 10.20	29.37 ± 8.49	= 0.09
HPDV (cm/sn)	55.88 ± 17.58	69.13 ± 27.41	= 0.06
CFR	1.75 ± 0.46	2.40 ± 0.80	< 0.005

BPDV: blsal peak diastolic velocity; HPDV: Hyperemic peak diastolic velocity.

## Discussion

The results of this study demonstrate that CFR and FMD were lower in CAD patients than control subjects and that CFR impairment correlates with the severity of peripheral endothelial dysfunction.

Increased blood flow is an important cause of endothelium-mediated vasodilation, which is reduced in pathophysiologic conditions [18]. Loss of endothelium-dependent dilatation in systemic arteries occurs in the early preclinical stages of CAD, being associated with known coronary risk factors, such as advanced age, male sex, hypercholesterolemia, hypertension, smoking and diabetes [19]. Endothelial function can be evaluated in coronary and peripheral circulation by monitoring the vasodilatation produced by the administration of endothelium-dependent agonist or by increased blood flow shears [20]. At the myocardial level, CFR is an important indicator of the significance of coronary lesions as well as other pathophysiologic conditions impairing coronary vasomotor function. The brachial circulation and the myocardial circulation differ in terms of microvascular architecture and resistance, pattern of blood flow, metabolic regulation and pathways that are activated to induce hyperemia [21]. Nevertheless, the assumption that endothelial dysfunction detectable in brachial arteries is a manifestation of systemic dysfunction, including the coronary circulation, can not be excluded because of the common underlying pathologic processes involved [22]. In fact, a close correlation between peripheral endothelial function and coronary endothelial function was demonstrated previously in patients with CAD [5]. Previous studies demonstrated that vasomotor dysfunction of epicardial coronary arteries is a predictor of cardiac events in patients without angiographic evidence of CAD [22]. In addition, it was shown that many patients with chest pain and normal vessels at coronary angiography have early atherosclerosis, as documented by intravascular ultrasound, reduced CFR and coronary endothelial dysfunction [23, 24].

### The relationship between brachial FMD and transthoracic CFR

Transthoracic CFR has been used to evaluate coronary microvascular functions and to evaluate conductive functions of epicardial coronary arteries. Several studies have demonstrated that endothelial function, determined with noninvasive modalities could predict cardiovascular events [25]. Brachial FMD correlates with measurements of coronary artery endothelial function and predicts future adverse coronary events [5]. However, it does not directly assess coronary circulation but, instead, serves as a surrogate of coronary vascular health. In our study we observed that transthoracic CFR correlates well with brachial FMD. Our results suggest that transthoracic CFR might be used as a surrogate of developing atherosclerosis (r = 0.232, P < 0.05).

### The relationship between carotid IMT and CFR

Takiuchi and others found an association between carotid IMT and transthoracic CFR which is generally regarded as indicative of coronary microcirculatory disturbance [26]. Our results are in agreement with these results with respect to the association between IMT and CFR (r = -0.403, P < 0.001). In published studies, FMD, carotid IMT and brachial artery pulse wave velocity showed stronger predictive power when used together as a surrogate of coronary atherosclerosis [27, 28]. Consistent with this finding when combined with brachial FMD and carotid IMT, transthoracic CFR might strengthen the combined predictive power of these non-invasive surrogates of atherosclerosis. Therefore, accuracy may be improved by the addition of the predictive power of transthoracic CFR.

### The relationship between carotid IMT and brachial FMD

In patients with atherosclerosis, a significant negative correlation between carotid IMT and brachial FMD has been reported [29]. In a published study Kobayoshi and colleagues suggested that carotid IMT significantly correlates with brachial FMD (r = -0.343, P < 0.001). In addition, they suggested that when taken together, FMD, carotid IMT and brachial artery pulse wave velocity have stronger predictive power as a surrogate of coronary atherosclerosis [27]. Our results are in agreement with these results with respect to the association between IMT and FMD (r = -0.211, P < 0.05).

The CFR measurement that reflects coronary microvascular function and endothelial function of epicardial coronary arteries by doppler echocardiography, as a cheaper and easy screening test may be used as a detection method in the assessment of major epicardial coronary arteries [7, 30, 31]. A CFR of < 2 may be evidence of severe coronary artery disease. In a recent report, Rigo [6] suggested that the use of CFR measurement as a sole diagnostic criterion has structural limitation. The Doppler assessment of coronary flow reserve has some limitations. The assessment of absolute blood velocity can be limited in some patients by the large incident angle between the Doppler beam and blood flow. However, calculation of the flow reserve allows assessment of flow patterns without the need for absolute values. More importantly, the velocity ratio is used as a surrogate of flow reserve. Flow within the coronary artery is not calculated because cross-sectional visualization of the vessel does not allow an accurate measurement of the diameter of the vessel. In addition, discrimination between microvascular and macrovascular disease could not be made by CFR measurement alone. However, CFR can identify mild to moderate stenosis (reduced flow reserve to subischemic levels) and coronary microvascular dysfunction (reduced flow reserve with angiographically normal coronary arteries). Microvascular dysfunction could lead to myocardial ischemia and cardiovascular disease [6]. Therefore measurement of CFR has value to predict future coronary events [32]. CFR may have a very useful role in our daily clinical practice even we think there is need for confirmation of the prognostic role of CFR in larger studies.

### Conclusion

Brachial FMD, Carotid IMT and transthoracic CFR evaluate different aspects of atherosclerosis as well as different sites of the arterial tree. Although each is regarded as being predictive of cardiovascular events, their combination might have more predictive value. However none of these measurements can be used to direct therapy for cardiovascular diseases with the exception of transthoracic CFR, which might be used to direct cardiovascular therapy and to follow the results of therapy. The results of the present study indicate that CFR is significantly lower in patients with CAD than control subjects. In addition, CFR impairment correlates with the severity of peripheral endothelial dysfunction. Follow-up studies are warranted to verify whether reduced CFR identifies a subset of patients who have CAD and who are at increased risk for cardiovascular events. At present, the impossibility of discriminating pathological behaviour of CFR due to microvascular or epicardial stenosis has represented a major limitation in the final diagnosis.
